# Dentistry as a free market in the context of leading policymaking

**DOI:** 10.1080/17482631.2018.1484218

**Published:** 2018-06-18

**Authors:** Bengt Franzon, Magnus Englander, Björn Axtelius, Björn Klinge

**Affiliations:** a Faculty of Odontology, Department of Oral Diagnostics, Malmö University, Malmö, Sweden; b Department of Social Work, Malmö University, Malmö, Sweden; c Department of Odontology, Division of Oral Diseases, Karolinkska Institutet, Faculty of Odontology, Department of Periodontology, Malmö University, Malmö, Sweden

**Keywords:** Public health research, public funding, welfare state, out-of-pocket payment, dental insurance, subsidy systems, dentistry, phenomenology, political decision-making, free market

## Abstract

The purpose of this study was to disclose the psychological meaning structure of dentistry as a free market within the context of leading Swedish policymaking. Following the criteria for the descriptive phenomenological psychological method data was collected from leading policy makers about the experiential aspects of dentistry as a free market within the context of a welfare state. The analysis showed that dentistry as a free market was experienced as a complex business relationship between buyers and sellers that transcended the traditional dentist and patient roles. The lived experience of the proposed business transaction was based on two inherently conflicting views: the belief in the individual’s ability to make a free choice versus the understanding that all individuals in a society do not have the ability or the means necessary to make a free choice. Dentistry as a free market within a welfare state, such as Sweden, can thus be seen as a persistent attempt to hold on to a compromise between two very distinctive political ideologies.

## Introduction

In Sweden, an adult patient’s right to choose his or her own dentist has never been questioned by government policymakers (Lindblom, 2004; Regeringen, 1973; Socialdepartementet, 2015). This distinguishes the dental health care section from the rest of the Swedish public health care system, even if the freedom of choice in terms of one’s dental care provider for patients has somewhat increased in recent years. The main difference between the health care system and dental health care is the nature of public funding (Sveriges kommuner och landsting, 2014). Dental care is mostly funded by the state, while the county councils fund the public general health care system. More importantly, patients’ self-financing of dentistry is still the economic rule of thumb, but there is a limit to the cost to the individual, making the state responsible when the patient’s dentist bill exceeds a certain amount or if the bill is connected to some sort of medical emergency. However, the Swedish economic support for adult dentistry is rather marginal compared to the overall public general health care system. Furthermore, it should not be forgotten that all paediatric dentistry is subsidized by public funding.

Sweden has a welfare system subsidized by the taxpayers in which adults’ teeth still do not qualify as a part of the body in the way that other body parts do. This makes it possible for dentists to become actors within a free market economy. Sweden is unique in the sense that it has both privately and publicly owned dentistry, each of which makes up approximately half of the total market (Privattandläkarna, 2017). General health care has been (and still is) dominated by publicly owned health care organizations. In the past in particular, this has created a market where private health care organizations have been considered as a complementary element (Sveriges kommuner och landsting, 2014). Given the complexity of such a context, one is rather curious about the psychology permeating policymaking, especially the meanings adopted in terms of dentistry as a free market. Hence, the purpose of this study was to explicate the contextual psychological meanings of Swedish policymaking in order to disclose a general, qualitative structure of the phenomenon: dentistry as a free market within the Swedish welfare state. Through an interview design, an in-depth exploration of contextual psychological dimensions was phenomenologically disclosed and rigorously described.

### Background

There is a long tradition of social democracy in Sweden serving as the underlying political ideology of a welfare state, even though a free market has been allowed to exist parallel to it, although it is controlled by the state (Åmark, 2011). Nevertheless, there has yet to be a significant political debate in Sweden with regard to dentistry as a part of the free market, regardless of the possibility of the reification (*Verdinglichung*) of the human body. There has also been a lack of ethical debates concerning the dilemma posed by the economic incentives for dentists in private practice, some of whom are also recognized as owners of dental companies and as primary caregivers of patients and who receive subsidies for some of their activities by the taxpayers. All this is in contrast to the rest of the public general health care system and public education where political discussions regarding privatization of welfare systems in Sweden have flourished (Socialdepartementet, 2016). Politically, there is little doubt that the question of dentistry as part of a free market would most likely reveal a tension between a socialist agenda of a welfare state for everybody and a liberal argument for the freedom of choice and a free market in order to assure competition which, it is argued, leads to high-quality care. Such a debate could find its primary foundation within the subject matter of philosophy and medical ethics. For example, the philosopher Edmund D. Pellegrino (1999, pp. 243–266) concluded:

Health care is not a commodity, and treating it as such is deleterious to the ethics of patient care. Health is a human good that a good society has an obligation to protect from the market ethos. It may be necessary to transcend politics in order to clearly see that the oral milieu is not just about cosmetics, but that this part of the body also belong to medicine and thus to the ethics of human life.

But is such a stance on the policymaker’s agenda?

Conducting qualitative interview studies with leading policymakers might present certain problems such as the interviewees embellishing reality, exaggerating their own importance, concealing unpleasant truths, etc. The elite, i.e., public figures with power and influence, usually have more to gain from presenting their view of reality than the ordinary citizen does (Hertz & Imber, 1995). The driving force behind this behaviour varies between individuals, but the political system and senior officials, as well as company culture, reward success and punishes failure (Bernhardsson, 2013). Society’s elite also differ from the average citizen with regard to social and educational background, i.e., they have a higher social status and a higher level of education (Putnam, 1997). Given this background, one should be very wary about regarding the qualitative interviews with leading policymakers as bearing the hallmarks of absolute facts. Nevertheless, a proficient, qualitative interviewer could make an interviewee comfortable in such a way that he or she reveals his or her lived experiences, making it possible to record material that later could be qualitatively analysed in order to disclose the meaning of the phenomenon being studied. Hence, the goal is to disclose the meaning of a phenomenon.

## Method

This study was attempting to answer the following research question: What is the psychological meaning of dentistry as a free market within a welfare state as seen from the context of leading policymaking? In order to answer the research question, a qualitative, phenomenological methodology, designed to explicate meanings, seemed an appropriate choice of methodology. The specific method utilized in this qualitative study was the descriptive phenomenological psychological method (DPPM) originally developed by Giorgi (1970, 2009).

### Ethical considerations

The research participants were leading policymakers in Sweden. The proposal was assessed by an ethical review committee at the Department of Odontology at Malmö University (No. OD 2014:407) and the Regional Ethical Board of Lund was questioned concerning the need for ethical approval for the study. No such ethical approval was deemed necessary. However, before any data were collected, the participants signed a consent form. The anonymity of the informants was secured when documenting the interview, as well as when the material was stored. The material was handled in a safe way to ensure the anonymity of the participants. The respondents have all read the transcribed interview and given their approval for the quotes used in the result section. They were free to withdraw from participation at any time and had the right to demand that their recorded interview was discarded.

### Data collection

Following the criteria for phenomenological data collection as described by Giorgi (2009) and Englander (2012, 2016), three in-depth, semi-structured interviews, each lasting approximately one hour, were conducted with senior policymakers.(The in-depth semi-structured interviews were conducted at the office of each of the respondents by BF. The interviews were semi-structured in the sense of the descriptive phenomenological psychological method (see Giorgi, 2009; also Englander, 2012) in that they followed one theme, that is, “Please describe a situation in which you experienced the phenomenon.” The interviews were analysed by B.F. under the supervision of M.E. The datasets supporting the conclusions of this article are available on request at the Department of Oral Diagnostics, Faculty of Odontology, Malmö University, Sweden.) Similar to purposive sampling and maximum variation sampling, in phenomenological psychological qualitative research the participants are strategically selected and variation ensured in order to study a contextualized phenomenon and not a population (Englander, 2012, 2016). Hence, in order to ensure the qualitative relation to the context of policymaking, variation among the research participants was necessary. The participants consisted of one senior politician with decisive influence on dental care, one senior chief official in a civil service department with dental-related activities, and one executive in private or public dental care. Gender was also taken into consideration.

### Data analysis

In the context of descriptive phenomenological psychological analysis, Husserl’s phenomenological method of the psychological was utilized in all steps of the qualitative method (Husserl, 1970; Langdridge, 2006). The eidetic reduction was used in steps 3 and 4. The purpose of the psychological reduction is to first bracket the existential claims of any given object as experienced by the research participants and consequently to gain a focus (a reduction) that enables one to disclose the psychological meanings as these present themselves within a particular context. The eidetic reduction is a critical process in which the essential psychological structure of the phenomenon is sought by a comparative analysis across the participants, although raised to the eidetic level as to constitute an essence as opposed to a commonality. The structure of the phenomenon is thus meant as an eidetic generalization and not an empirical generalization. The phenomenon is not considered an empirical object, but instead what Husserl (1977) termed an *ir-real* object; i.e., a form also known as a phenomenon and constituted by meanings, supported by the epistemic relation known as intentionality.

Following DPPM as described by Giorgi, the researcher, after having adopted the psychological reduction, (1) reads the entire set of data over and over again in order to get a sense of the whole; (2) divides the data set into workable meaning units; (3) describes the psychological meanings in each meaning unit on the basis of the participants’ expressions (here also utilizing the eidetic reduction); and (4) discloses, using the eidetic variations within the psychological reduction, the psychological meaning structure of the phenomenon under investigation. The results are descriptive in the sense that the structure is not forced into a theoretical framework, but constitute a form based upon meaning. However, since language is used to articulate the description and generative meanings apply to language, further hermeneutic analysis could be suggested, but such an analysis was not conducted in this study.

## Results

Before presenting the general results, it is essential to point out that the results are not to be read as an empirical generalization, i.e., as a summary or commonality within three cases, but instead as a phenomenological description (using the method of the psychological reduction) of essential, psychological meanings disclosed within the context of the three cases and hence constituting an eidetic generalization (Giorgi, 2009). First, the general (psychological meaning) structure of the phenomenon will be presented. This is then followed by the constituents (i.e., context dependent parts), making up the necessary, qualitative structure of the phenomenon.

### The general psychological meaning structure of the phenomenon

The general psychological meaning structure of the lived experience of dentistry as a market, as experienced within the context of senior policymaking in government, can be described as follows:

In the context of development in dentistry and senior policymaking in government, government agencies, and dental business, dentistry is understood to be a complex business relationship between buyers and sellers that transcends the traditional dentist and patient roles. The traditional roles of the dentist and the patient still remain but these roles end up in the background within the context of a business exchange. The lived experience of the proposed business transaction is based on two inherently conflicting views: the belief in the individual’s ability to make a free choice versus the understanding that all individuals in a society do not have the ability or the means necessary to make a free choice. The awareness that the individual’s freedom to choose a provider and specific health care content is based on the assumption of and belief in the individual’s own ability to make a choice in which he or she is able to use his or her financial capacity and knowledge background as a customer. The understanding and awareness of individuals’ varying financial capacity and knowledge background sets up a compensatory type of thinking. This is expressed in the need for community involvement in order to protect vulnerable groups against economic inequality and disadvantages, in terms of the knowledge demanded to make an informed, free choice. Patients’ vulnerability is regarded as amplified in the situation of having a free choice. In the context of the patient/dentist professional relationship, the dentist is seen as an authority figure, i.e., as having an advantage in terms of knowledge, over the patient. The solutions to these conflicting views are believed to be found in state subsidies and strict regulations, motivated by the attempt to achieve a functioning dental market for the majority of citizens.

### The constituents

The four necessary constituents making up the general structure can be identified as follows:

1. The figure-ground of the seller-dentist relation
2. The conflict between the idea of a free choice in relation to a free market and the lack of necessary financial ability and knowledge background
3. The strive to protect against economic inequality
4. The belief in state subsidies and strict regulations as the solution for a working market.

#### Constituent 1: the figure-ground of the seller-dentist relation (Figure 1)

The first constituent identified within the data was that the traditional role of the dentist and the patient ended up in the background (although remained intact) within the context of a business exchange. Patients’ vulnerability was seen as amplified within the alleged situation comprised by a free market choice and the background of the patient/dentist relationship, in which the dentist was seen as having the authority, in terms of knowledge, over the patient. For example, the research participants, i.e., Participant 1 (P1), Participant 2 (P2) and Participant 3 (P3), stated:

As a rational person, understanding that it is for my own good, I am comfortable in taking care of myself without anyone telling me what to do. Dentistry has, in some sense, succeeded in creating this behaviour. (P1)

Quality is very much an experience because lack of a clear definition. It is an experience. The experience is based on the patient’s expectations created by the market and by the service provider (the dentist) … I think that decisions taken by patients primarily, like most decisions, are emotionally based. Quality for patients is the impression of feeling content and being in safe hands. (P2)

Dental examination that leads to a cost estimate and the opportunity to obtain a second opinion, even though you have to pay it yourself, are requirements for making very good informed choices … The problem is that you are not able to evaluate the quality of treatment. (P3)

**Figure 1.**
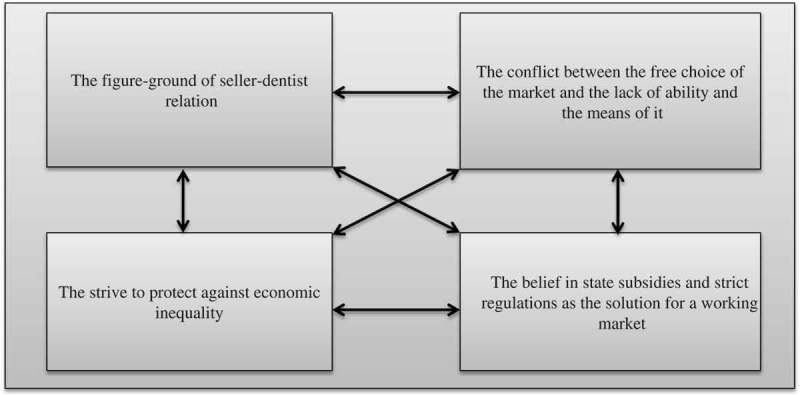
The general psychological meaning structure of the lived experience of dentistry as a market, experience within the context of senior policymaking.

#### Constituent 2: the conflict between the free choice of the market and the lack of ability (financial ability and knowledge background) and means for it (Figure 1)

The second constituent identified within the data was the conflict between the belief in free choice and the understanding that some citizens actually lack the ability to make an informed choice and/or do not have the means necessary to be an actual player in a free market. The reasoning underlying this, as constituted in the data, was that a strong awareness of such limitations became apparent for the policymakers. For instance, the policymakers interviewed in this study were all aware that not all patients are able to use their opportunities of making free choices. Two crucial factors were identified: (1) the individual financial circumstances, and (2) the individual’s ability to understand the system of a free market. In turn, some individuals were seen as unable to use their power and strong position as consumers and, as a consequence, the opportunity to make a better choice. Both factors may apply separately but also coincide within the context of the inability to make informed choices. For example, the participants stated:

The patients have knowledge gaps. I believe that patients do not use their mandate and means. Patients live in the belief that there is great similarity between dentistry and the more regulated health care system. They believe that someone has taken care of their rights in terms of rates, that they get the offers and options that they should have. (P1)

I think it’s good that we have the framework for a minimum standard. As a patient, you know that there are rules and regulations no matter which dentist you choose. At least there are authorities to ensure this. (P2)

There is a market when I as an individual have the opportunity to choose. The great challenge is to create a system where the consumer can make an informed choice. (P3)

#### Constituent 3: striving to protect against economic inequality (fig.)

The third constituent identified within the rationale of the phenomenon was the endeavour to protect against economic inequality in order to create a dental market within the context of a welfare state. To guard against the disadvantage and the prejudice that stems from the idea that a dental status also reflects a social status, dental care subsidies were motivated within this particular welfare rationale. Subsidies were seen as the way to reduce social inequalities and create a fair system whilst at the same time maintaining the fundamental idea of dentistry as a market. For example, the participants stated:

On a group level we cannot see any alarming differences or increases in differences, nor any challenges in dental health. The challenges are due to other factors such as increased immigration. This is quite important as a political mission. (P1)

For patients younger than 65 it was expensive using the dental market prior to 2008. The reform of 2008 was created to give everybody the opportunity to use the market. The state chose to subsidize dentistry and make it as fair as possible without causing the failure of the market idea or creating conditions for exploiting the system, i.e., a generous system. (P2)

To avoid that the status of teeth becomes a class issue, there must be public control of the dental sector trough subsidies and regulations. To get access to the subsidy system, patients and dentists must follow the rules and prioritization principles. (P3)

#### Constituent 4: the belief in state subsidies and strict regulations as the solution for a working market (Figure 1)

The fourth constituent disclosed within the rationale was the belief in the need for strict regulations and state subsidies to achieve a working free dentistry market within a welfare state. There is a strong belief that a free market must be balanced by mandatory rules and control of compliance to function within welfare systems; where the state formulates the rules, and regulates the compliance with the rules and where the dental sector respects them. For example, the participants stated:

It’s a market that works well in many aspects. It works less well in other aspects. If the government decides that there are some parts of this market that is not working, it will intervene. (P1)

It would be nice if the state does not interfere in the market in general terms. Like in the old times when “you had to go to the Minister of Finance to be able to make an investment”. As the system is designed today it is not hampering the market. I think it’s fine with existing regulations and that there are authorities such as TLV (The Dental and Pharmaceutical Benefits Agency), IVO (The Health and Social Care Inspectorate), Strålsäkerhetsmyndigheten (The Swedish Radiation Safety Authority), etc., that control compliance. (P2)

To ensure a working market, the professionals have to dare to expose themselves for control. There are very few who actually get convicted. And yet it is a regulated occupation with ways to impose sanctions. It is a job with responsibility, and with that comes means for sanction. (P3)

## Discussion

### Method

The delimitations and the limitations of this study are intricately related. The delimitation to focus on psychological meanings and the aim to discover the psychological meaning structure of the phenomenon suggested a phenomenological investigation. Hence, there was no attempt at finding causal relations but instead the focus was on finding meaning relations based on intentionality (Englander, 2016). Drawing from the lived experience of empirical subjects (i.e., the research participants), the analysis entailed a phenomenological scientific methodology as opposed to a philosophical. However, such delimitation does not automatically entail a limitation in terms of also pursuing a philosophical study in which the phenomenon could be broadened to a phenomenological inquiry into the meaning of policymaking in a welfare state, in which a free market economy is maintained. In addition, by not seeking a causal relation, the results are limited to the realms of presentational evidence of the psychological meanings as these appeared in the raw data.

Generalizing the results of a qualitative study always seems to be a critical topic for a discussion of methodology. One has to remember that the three participants used in this study are not equivalent to three participants in a quantitative study, in which statistical representations of a given sample are being critically evaluated. Rather, sampling strategies in phenomenological scientific research are better compared to the use of purposive sampling in qualitative research or exceptionally clear cases in neurological research or even the so-called thought experiments in philosophy, in which specific depth is sought about something very specific; in the case of this particular study, a contextualized phenomenon (Lincoln & Guba, 1985). Hence, the aim was not to make general knowledge claims about a sample and how it is representative of a particular population. Instead, a phenomenological psychological study is seeking the general structure of the phenomenon. The eidetic generalization of the structure constituted by the meaning of the phenomenon will always transcend the population with which it is associated, indicating that the meaning of the phenomenon is not *in* the population (Englander, 2012, 2016; Giorgi, 1997). Also, and to further clarify, to draw from participants’ lived experience does not indicate a subjectivism or relativism, because the meaning of the object (i.e., the phenomenon) is sought by transcending (using the methods of the psychological and the eidetic reduction) its dependence on the subjective act of the research participant. The research participants of this study were strategically selected and variation ensured in order to study a contextualized phenomenon and not a population.

Eidetic generalizations, not empirical generalizations, are thus sought. In addition, the results must later (in the discussion) be able to relate to the social and historical context in a meaningful manner (Davidson & Cosgrove, 1991, 2003; Giorgi & Giorgi, 2008). Hence, the eidetic reduction (as part of the step 3 and 4 analysis, see Figure 1) ensures that general knowledge claims have been critically assessed in relation to the phenomenon under investigation (Giorgi, 2009; Wertz, 2010).

### Results

A tentative conclusion based on the results suggests that today’s policymakers are managing a dental heritage using ad hoc solutions in order not to disrupt the market too much, but rather to align their decisions with the idea of a welfare state. Reasoning about welfare, the market, freedom of choice for the patient, and so forth, seem to be a way of making sense of policymakers’ own ideologies in relation to the inevitable historical conditions and facts. The development of dentistry as part of a welfare state has been characterized by gradual implementations of benefits for parts of the population. The sociologist Esping-Andersen identifies the Swedish welfare system as a Social Democratic Party system and claims that politics is an important factor in explaining the growth of different social regimes (conservative, liberal or social democratic; Åmark, 2011). The Social Democratic Party had the advantage of ruling over long consecutive periods during the twentieth century, which enabled it to set the agenda and to establish alliances with the private sector. Korpi and Palme identified how the struggle between labour and capital resulted in the welfare system Sweden has today (Åmark, 2011). Slowly but surely a welfare system has been implemented on the basis of a free market, even though the market and dentistry were almost never mentioned in the public domain until the mid-1990s (Socialdepartementet, 1998). Considering that background, it is now possible to situate the results within the context of the welfare state and policymaking. The figure-ground of seller and dentist, in which the seller constitutes the figure in this perceptual relation, was then to be more easily allowed to emerge and to become public within the political context that developed in Sweden in the 1990s, a period in which the persistent rule of the Social Democratic Party was weakened (Jönsson & Karlsson, 1994; Karlsson, 2005).

Nevertheless, the main aim of the dental insurance system was to “make good dental care financially accessible to all citizens” (Regeringen, 1973). Thus, dental care became a part of the welfare state in which the state plays a key role in the protection and promotion of the economic and social distribution of wealth. This is combined with a public responsibility for those unable to provide for themselves the minimum requirements for a good life. In other words, the dental conditions, not the economic situation of the citizen, were to determine the extent of the state subsidies for dental care. Lingering within the policymakers’ experience there is still, as the results indicate, a conflict between a free choice of the market and the patients’ lack of ability and means to make such a choice. At the same time, within the context of leading policymaking, there is an endeavour to protect against economic inequality, and thus protecting the socialist agenda. Even though the various players and the function of the market have been handled in different ways over the years, the patient’s right and opportunity to choose a dentist have always been fundamental to Swedish dentistry and have never been questioned (Socialdepartementet, 2015).

The perfect market is characterized by a balance between supply and demand (dentists and patients), and a balance in the relationship between the customer and the seller of a product or service. In dentistry, as in most markets, this balance is often not achieved (Jönsson & Karlsson, 1994). When it comes to balancing the socialist agenda with the free market, one can see in the results that the belief in state subsidies and strict regulation is a way for policymakers to situate the idea of welfare and a free market working as an integrative system. In broad terms, such a balancing act seems to work. A report made by the Swedish Government (Socialdepartementet, 2015) showed that the Swedish dental market is characterized by high market penetration, low customer mobility and high customer satisfaction (i.e., 60–80% of the population visit the dentist within a 2-year period, and most patients have a long relationship with their dentist). Furthermore, dental care has the highest customer satisfaction by far in comparison to all sectors of Swedish health care (Svenskt kvalitetsregister, 2015). The social philosophical question seems ultimately to be about freedom of the individual versus the collective responsibility of the state.

### Further studies

This study describes the phenomenon of dentistry as a free market within the context of leading policymaking. Therefore, further research is needed to understand the phenomenon within the context of being a patient and a practitioner.

### Conclusion

Therefore, from the perspective of policymaking, dentistry as a free market within a welfare state such as Sweden can be seen as a persistent attempt to hold on to a compromise between two very distinctive political ideologies. The compromise seems to be taken for granted, perhaps in order not to disrupt a system that actually seems to be working. To never seriously question dentistry as a free market seems like a remarkable historical achievement, considering that Sweden has had a rather consistent rule of social democracy for more than a century. Hence, the context of policymaking continues to set parts of the oral milieu apart from the rest of the human body (that is covered by Swedish public general health care) and tries to maintain an oral reification as the foundation for dentistry. The question is whether or not a public debate can pull the teeth out of the market and put them back into the body. But perhaps there is also a possibility that a public debate could go the other way and show how a functional dentistry system, with high customer satisfaction and high market penetration, can lead the way for policymakers in an attempt to find a new solution for what seems to be a functionally declining Swedish public general health care system.
